# Evaluation of the Efficacy of UV-C Radiation in Eliminating Microorganisms of Special Epidemiological Importance from Touch Surfaces under Laboratory Conditions and in the Hospital Environment

**DOI:** 10.3390/healthcare11233096

**Published:** 2023-12-04

**Authors:** Anna Różańska, Monika Walkowicz, Małgorzata Bulanda, Tomasz Kasperski, Edyta Synowiec, Piotr Osuch, Agnieszka Chmielarczyk

**Affiliations:** 1 Department of Microbiology, Faculty of Medicine, Jagiellonian University Medical College, Czysta str. 18, 31-121 Kraków, Poland; malgorzata.bulanda@uj.edu.pl (M.B.); agnieszka.chmielarczyk@uj.edu.pl (A.C.); 2 Department of Metal Working and Physical Metallurgy of Non-Ferrous Metals, Faculty of Non-Ferrous Metals, AGH University of Krakow, al. A. Mickiewicza 30, 30-059 Krakow, Poland; monika.walkowicz@agh.edu.pl (M.W.); piosuch@agh.edu.pl (P.O.); 3 John Paul II Specialistic Hospital, Prądnicka 80, 31-202 Kraków, Poland; e.synowiec@szpitaljp2.krakow.pl

**Keywords:** infection control, UV-C disinfection, ESKAPE, *Candida auris*

## Abstract

Introduction: Healthcare-associated infections in the post-pandemic era are as important as they were before COVID-19. The dominant route of transmission of microorganisms in health care units is the contact route, for which hand hygiene is of cardinal importance, but also effective disinfection of touch surfaces. Traditional disinfection based on chemical compounds is sensitive to human errors. Therefore, a valuable supplement to it can be contactless disinfection methods, including the use of UV-C. The aim of the study was to assess the effectiveness of UV-C radiation in eliminating selected, most important pathogens of particular epidemic importance from surfaces made of various materials: stainless steel, plastic and glass, most often found in hospital conditions. Material and Method: In laboratory conditions, the study was conducted using bacterial strains of great epidemiological importance and *Candida auris*. In hospital wards, samples were taken before and after disinfection for comparisons of the composition and quantity of bacteria. In laboratory conditions, carriers made of steel, plastic and glass were contaminated with a bacterial suspension with a density of approx. 0.5 McFarland, and then the density of persistent microorganisms was assessed after 10 min of UV-C irradiation. Results: The high effectiveness of UV-C radiation in eliminating bacteria contaminating touch surfaces in hospital wards and in laboratory conditions has been confirmed. The elimination efficiency in laboratory conditions was slightly lower (statistically insignificant) on the plastic surface, which is probably related to subtle differences in the thickness of the contaminating layer. Hydrophobic properties and the smallest suspension diameter were confirmed for the tested plastic carriers. Conclusions: UV-C disinfection is a desirable element to support traditional, chemical methods of disinfection in hospital conditions, effective against multidrug-resistant bacteria and *C. auris*.

## 1. Introduction

Healthcare-associated infections are one of the main problems of modern medicine. These infections are found in different patient populations at varying rates. Etiologic agents depend on the clinical form and the unit in which the patients are staying. However, the general trend is that the incidence of drug-resistant bacterial strains is increasing and that the hospital environment is frequently contaminated with them [1,2,3,4]. These microbes are able to survive on inanimate surfaces, including medical equipment, for prolonged periods of time. The frequency of how often the staff touch contaminated surfaces correlates with the contamination of hands and protective gloves [5]. If patients are hospitalized in rooms, which were previously occupied by other patients colonized or infected by, e.g., MRSA strains, the risk of infection or colonization with these bacteria increases significantly [6,7]. It has been confirmed that the implementation of effective decontamination of patient rooms and the introduction of contactless disinfection methods lead to a decrease in the number of infections in subsequent patients [8]. Analysis of the available research results shows that contamination of surfaces with pathogenic bacteria in hospital wards varies. As regards MRSA, from 1 to 27% of surfaces were reported to be contaminated with the strains, and for *Acinetobacter*, it was from 3 to 50% in epidemic outbreaks, whereas for *C. difficile*, in rooms with patients with confirmed infections, the percentage was from 2.9 to 75%.

Despite frequent surface contamination, the number of CFU per cm^2^ of the surface does not usually exceed 10. In a Polish study from 2015, bacterial growth was demonstrated in 46.4% of samples taken from touch surfaces in wards of various types in three hospitals in Małopolska [9]. As regards the number of CFU per cm^2^, a relatively modest growth was recorded. In the vast majority of cases, i.e., 39.1–62.5%, depending on the hospital, the number of CFU per plate was within the range of 4–10 CFU.

It would therefore seem valid that traditional decontamination processes, including chemical disinfection, are sufficient to effectively eliminate microorganisms living on surfaces in healthcare facilities. However, the effectiveness of decontamination, in particular chemical disinfection, is affected by a number of factors, e.g., haste, which may result from cleaning staff shortages and a lack of time, a preparation that is not adjusted to the type of surface/contamination, and others. It has been shown that even over 50% of surfaces might be missed during cleaning [7,10]. The COVID-19 pandemic also demonstrated that, even in highly developed countries, there may be shortages of chemical disinfecting agents [11].

Due to the already discussed conditions concerning traditional cleaning and disinfection, it is important to introduce additional disinfection methods into everyday practice which would be less dependent on human errors, quick, easy to use and effective [12,13], e.g., devices enabling contactless disinfection with UVC radiation. UVC radiation at 254 nm was confirmed to have antimicrobial properties, resulting from the destruction of DNA and RNA; however, its effectiveness, similar to any other method of decontamination, depends on various factors. The aspects to be mentioned here are the distance of the surface subjected to disinfection from the source of radiation, the wave length, operation time, shading, which can be given by furniture, and also organic pollutants [14,15]. Hence, in principle, UVC disinfection should be preceded by cleaning and undoubtedly depends on technological capacities of individual devices. Furthermore, studies on different disinfection methods have shown that their effectiveness may vary slightly not only between different groups of microorganisms, but even between strains within a given species [15,16,17].

The aim of this study was to determine the effectiveness of UVC radiation disinfection using the technology applied in the OCTA UV-System for selected strains of bacteria of significant epidemiological importance and fungi from the genus Candida. The system was employed under laboratory conditions and in a hospital environment.

## 2. Material and Method

### 2.1. Evaluation of the Efficiency of Eliminating Microorganisms of Particular Epidemiological Importance under Laboratory Conditions

This study employed a method consisting in contaminating plates made of various materials, from now on referred to as carriers, with a specific volume of bacterial suspension of known density and subjecting them to UVC radiation disinfection with the use of OCTA UVC robots. Three types of test plates were employed for the study: they were made of stainless steel, plastic, and glass, cut into squares with 2 cm sides. Materials, which the test plates (carriers) were made of, were selected in terms of representativeness as regards hospital equipment of various types, in order to reflect real conditions as much as possible. The effectiveness of disinfection was tested for the strains within the ESKAPE group (*Enterococcus faecalis*—EF, *Staphylococcus aureus*—SA, *Klebsiella pneumoniae*—KP, *Acinetobacter baumannii*—AB, *Pseudomonas aeruginosa*—PAR, *Enterobacter cloacae*—ECLO) and *Candida auris*—CA.

All strains came from the collection of the Department of Microbiology of the Jagiellonian University Medical College and were isolated from infections in hospitalized patients. The efficiency of disinfection with the use of the OCTA UV-System was tested for the device operation time, which is 10 min. The research was carried out in a room with an area of 27.45 m^2^ (room height 3.8 m).

**Figure 1:**
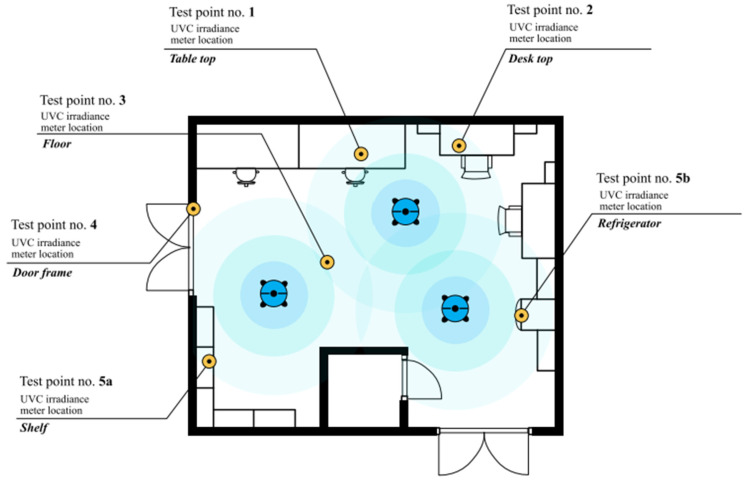
Plan of the room where the effectiveness of surface disinfection using the UV-C method with the use of OCTA robots was tested.

The carriers were placed in five different spots of the room—Figure 1.

Subsequently, OCTA UVC robots were turned on for 10 min, and then the bacterial cells that survived on the carriers were recovered, their numbers were determined and the degree of reduction in relation to the initial density of the bacterial suspension was assessed. The robots emit UV-C wave of 253.7 nm length.

### 2.2. A Detailed Procedure for Testing the Effectiveness of Eliminating Strains from Contaminated Surfaces

The six bacterial strains were grown on TSA solid medium (Tryptic-Soy Agar) at 37 °C overnight and under aerobic conditions.

1. The next day, the test strain was collected with the eye of the loop and suspended in physiological saline. The bacterial suspension density was adjusted to 0.5 McFarland using a densitometer. An amount of 100 µL of the prepared suspension was taken and added to 900 µL of the TSB (Tryptic-Soy Broth) growth broth. To obtain a test suspension density, i.e., in the growth broth, around 2−4 × 10^6^ CFU/mL was assumed.
2. In order to accurately determine the density of the suspension, the obtained solutions were plated by serial dilution on solid TSA medium and cultivated for 24 h under aerobic conditions at 37 °C, after which the obtained bacterial colonies were counted and the density in colony forming units (CFU) was determined per milliliter.
3. For each test carrier and for each strain, five test samples and one control were prepared. The samples were prepared by applying 50 µL of the suspension prepared as described in point 2 to each of the carriers and then this volume was dispersed with a pipette tip and left to dry.
4. After the suspension had dried, the test carriers were placed at five test points in the room where the robots were run for a specified period of time, i.e., disinfection was performed, and the control carriers were moved to the adjacent room, so that they were not exposed to UVC radiation. The layout of the test plates is shown in Figure 1.
5. After the disinfection process was completed, the previously applied material—bacterial suspension—was collected from each carrier in order to assess the number of surviving bacterial cells. ESwab^®^ flocked swabs (Copan, Murrieta, CA, USA) were used for this purpose. Each of the test carriers and control ones were wiped with a swab wetted in the substrate, then the swab was placed in a tube with 1 mL of liquid medium. The tube with the medium and swab was then vortexed, and the washings were plated by serial dilution onto TSA solid culture medium.
6. Petri dishes with seeded material were placed in an incubator, and after 24 h of cultivation (at 37 °C) removed from the incubator, and the grown bacterial colonies that survived the disinfection process were counted (for controls, colonies were counted to accurately establish the density of the suspension as a reference to the reduction rate estimate).
7. The number of obtained colonies was converted to density in terms of CFU/mL, and for each test sample, the percentage reduction was also calculated with respect to the density of the suspension in the appropriate control carrier.
8. The initial bacterial suspension was also inoculated to determine its exact density (CFU/mL).

### 2.3. Assessment of the Effectiveness of Eliminating Microorganisms under the Conditions of Hospital Units

The study was conducted in six rooms of the hospital located in: I Department of Anesthesiology and Intensive Care (ICU I), III Department of Anesthesiology and Intensive Care (ICU III), Department of Vascular Surgery with the Subdepartment of Endovascular Procedures (VS) and Department of Heart, Vascular Surgery and Transplantology (HVT) and Cardiosurgery. The ESwab^®^ locked swabs were used for taking samples from various types of touch surfaces in each ward. The surfaces were divided in terms of the type of material they were made of (i.e., plastic, metal and glass), maintaining the same assumptions as in the case of laboratory conditions. For this purpose, a detailed swab collection protocol was prepared in order to standardize the entire procedure. According to the protocol, about ten samples were taken using of swabs from the chosen surface of about 10 cm^2^, before and after disinfection. The items (surfaces) for sampling were chosen by infection control and prevention nurses. There was a diversity of types of surfaces in the rooms, but before and after disinfection in a given room, samples were taken from the same items. Time of disinfection was settled automatically by the UV-C robots, depending on the patients’ room’s diameters. Rooms for testing had to be (due to direct UV-C exposure) empty, so in the time for performing the study, rooms which could be available for cleaning between patient discharge and admission were chosen. However, epidemiological surveillance run at study hospital shows that patients in all rooms are exposed to a similar risk of infection.

Swabs were taken from electrical sockets, bed frames, drip infusion stands, door handles, toilet seats, bedside tables, anti-bedsore mattress pumps, stethoscopes, washbasin taps, mirrors, door glass, etc. A detailed summary of the surface types in each unit was presented prior to decontamination (cleaning or UVC disinfection) as well as after cleaning and UVC disinfection. The smears taken with the use of ESwab^®^ flocked swabs were inoculated on growth media, incubated at 37 °C for 24 h, and the number of colonies was counted up. The cultured colonies were isolated and subjected to species identification with MALDI-TOF.

The obtained numbers of colonies isolated before cleaning were compared with the number of colonies obtained after cleaning and with the number of colonies obtained following UVC disinfection, which made it possible to assess the percentage reduction in the number of CFUs isolated in samples collected at individual stages.

All growth media used in the study were prepared in the laboratory of Chair of Microbiology, and went through internal validation and control, both for sterility and nutritious properties. For each strain tested and each type of carrier, controls were carried outin addition to the control of initial bacterial suspension density in order to eliminate potential bias connected with losses of bacterial cells due to swabbing.

In the laboratory part of the study, we performed pilot tests with disinfection three times—6 min, 10 and 20 min. However, pilot results did not show substantial differences, so for further in depth analysis, we choose the time 10 min as optimal for practical reasons and in connection with efficient elimination of microorganisms. In the clinical part of experiment, the duration of each disinfection procedure was settled automatically by the robot. However, these procedures also can be burdened with some potential biases, and of course, direct exposure of UV-C may only occur in rooms without the presence of people.

### 2.4. Methods of Surface Wettability Characterization

In order to determine surface wettability of tested materials and its relation to antimicrobial efficacy, the contact angle was measured utilizing the sessile drop method.

Wettability of the materials was determined by placing 2 μL droplet of a demineralized water at 20 °C and with a relative humidity of 50% in a horizontal microscope with a protractor eyepiece and environmental chamber (KRÜSS DSA-25, KRÜSS, Hamburg, Germany). Ten measurements of the contact angle were made for each of the samples, and the results were averaged.

### 2.5. Statistical Analysis

Basic descriptive measures such as median and standard deviation of the level of reduction were calculated. Statistical analysis was carried out on the basis of both load logarithm as well as % of reduction after disinfection. Determination of the significance of differences between the site of disinfection or the type of material and a reduction in the load of individual strains depending on the site or material was carried out using non-parametric tests, and the distributions in the groups were compared using a Bonferroni-corrected Mann–Whitney U test. The results were considered significant at *p* ≤ 0.05.

## 3. Results

### 3.1. Evaluation of the Efficiency of Eliminating Microorganisms of Particular Epidemiological Importance under Laboratory Conditions

All microbial species showed a reduction in CFU/mL; the percentage drop with regard to this reduction was from 59.3 to 100%, taking into account bacterial strains, type of carrier and location. In logarithmic terms, the reduction ranged from 1 to 6 logs.

Analysis of the reduction level taking into account all locations for given microorganisms and the type of carrier showed a slightly higher reduction in the case of glass and a lower reduction in the case of plastic (Table 1). When glass was used as a carrier, four of the microbes tested were fully eliminated (100%), and a reduction of three or more logs was reached in 70% of the cases. For plastic, here, 60% of the samples did not reach a reduction of three or more logs.

Statistically significant differences were observed between the type of material and the logarithm of bacterial load after disinfection for the steel–plastic material pair, *p* = 0.041; for steel, the reduction was greater by 0.74 logs.

This correlates with the results of wettability depending on the contact angle of the droplet.

Taking into account microorganisms, the highest efficiency was observed for *Pseudomonas aeruginosa.* Regardless of the type of carrier material or location, the reduction was from 99.98 to 100%, and it was always a decrease of at least three logs CFU/mL. Other species that showed high colony reduction include *Klebsiella pneumoniae* (only one sample failed to drop by three logs), *Enterobacter cloacae* and *Staphylococcus aureus* (in these species, 3 out of 15 samples did not reach a drop of three logs). The remaining samples tested showed a reduction in the number of colonies of one log at most.

A slightly higher, but still unsatisfactory, reduction concerned *Enterococcus faecalis,* but only for two test points (one on glass and one on steel) was a drop of more than three logs achieved. Within the bacilli, the smallest reduction was present in the case of *Acinetobacter baumannii*, especially when a plastic carrier was used; in this instance, the reduction did not exceed three logs for any of the sites studied.

We observed significant statistical differences in the efficacy of UV-C disinfection across three different surfaces. On plastic (Table 2), there were significant differences between *Acinetobacter baumannii* (AB) and strains like *Pseudomonas aeruginosa* (PAR), *Candida auris* (CA), and *Klebsiella pneumoniae* (KP) (*p* = 0.045), and between *Enterococcus faecalis* (ENT) and strains such as PAR, CA, KP, *Staphylococcus aureus* (SA), and *Enterobacter cloacae* (ECLO) (*p* = 0.003). On glass (Table 3), notable disparities were found between AB and SA, CA, and PAR (*p* = 0.008), and between ENT and SA, CA, PAR, and KP (*p* < 0.001). On steel (Table 4), significant differences were observed for ENT in comparison to PAR, SA, CA, and KP (*p* = 0.025). These results emphasize that the surface material plays a crucial role in the effectiveness of UV-C disinfection, indicating the need for surface-specific disinfection strategies in healthcare environments.

Looking at reduction in relation to the location of the carrier in the room (Figure 1), the greatest effectiveness was observed for the samples placed in point no. 4, regardless of the bacterial species or carrier. A reduction of three or more logarithms was present in 71% of the samples. The smallest efficiency was found in the samples placed in point no. 5, where a reduction of three or more logarithms occurred only in 33% of the samples.

As regards the test point, statistically significant differences were observed on the basis of the logarithm of bacterial load between points 2 and 1, 4, and 5 (*p* < 0.001), point 3 and 1, 4, and 5 (*p* = 0.006), as well as point 2 and 1, 5 (*p* = 0.012).

Detailed results showing the reductions according to microorganisms, type of carriers and location are shown in Table 2, Table 3 and Table 4.

### 3.2. Evaluation of the Effectiveness of Eliminating Microorganisms in the Hospital Environment

The aggregate data were subjected to two analyses, separately for the four units in which cleaning preceded UVC disinfection and the two units in which UVC disinfection was carried out without previous cleaning. In all the rooms from the first analysis, the majority of samples taken before cleaning gave a positive result, i.e., bacterial growth was confirmed: in the room in the Vascular Surgery Ward this was eight out of nine samples, in room HTX I (Heart Transplantation) ICU I, this was nine out of eleven samples, in the room in the III Intensive Care Unit, this was five out of seven samples, and in the last one, this was seven out of ten samples (Table 5).

The number of colonies obtained from all sampling sites was from 105 to 320.

After cleaning, a reduction in the number of bacterial colonies was found to amount to from approx. 8% to approx. 90%, while UVC disinfection carried out after cleaning led to a reduction in the number of bacterial colonies of from approx. 96% to 100%—detailed data are contained in Table 5.

In the rooms from the second analysis, most of the samples taken before cleaning gave a positive result, meaning that bacterial growth was confirmed: in one room in the cardiosurgery ward this was nine out of ten samples, and in the other—in the heart, vascular surgery and transplantology recovery room—this was ten out of eleven samples. The number of colonies obtained from all sampling sites was from 213 to 362.

Following UVC disinfection, there was a reduction in the number of bacterial colonies of from 80% to 95%.

The bacterial species isolated from the collected samples represented mainly bacteria living in the environment, or forming the skin microbiome; however, in each of the hospital rooms, pathogenic species, common etiological agents of infections, were also isolated, including the ones present in epidemic outbreaks, such as *E. faecium*, *P. aeruginosa*, *E. faecalis*, *Pseudomonas fulve* and *A. baumannii*. In one of the rooms, following cleaning, the presence of *P. oryzihabitans* was confirmed, while the remaining species were bacteria typical of the environment and human skin. After UVC disinfection, two rooms showed no bacterial growth, and the others demonstrated only representatives of environmental bacteria and the skin microbiome.

### 3.3. Surface Wettability

**Figure 2:**
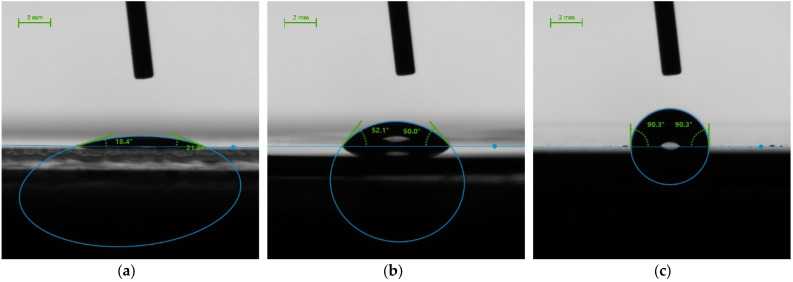
Contact angle measurements of test materials: (**a**) glass, (**b**) steel, (**c**) plastic.

All tested materials (glass, steel, plastic) were subjected to contact angle measurements. Test materials were placed in the environmental chamber set up on 20 °C and 50% relative humidity. Precisely controlled environmental conditions allowed to conduct measurements of evaporation time of a water droplet of 2 μL volume. Measurement results are shown in Table 6. Photographs of the initial droplets put on the particular materials are shown on the Figure 2.

The results of surface wettability indicate that the most wettable, i.e., the smallest contact angle at the level of approx. 20°, was glass. The contact area of the water droplet with this moderately superhydrophilic material was the largest and the evaporation time was the shortest at 13 min. The least wettable, i.e., the largest contact angle, was noted for plastic (Θ = 90°). In the case of this hydrophobic material, the evaporation time was the longest and lasted almost 1 h. Steel has demonstrated hydrophilic properties in tests. The wetting angle was about 50° and the evaporation time was 33 min.

## 4. Discussion

The present laboratory study confirmed the high effectiveness of UVC radiation in eliminating bacteria and *Candida auris,* which are etiological agents of nosocomial infections, including the drug-resistant and spore-forming microbes.

The method employed to assess the density of the bacterial suspension is subject to an error of approx. one log; therefore, it is justifiable to determine the absolute density of the suspension, as well as the relative presentation—the percentage of reduction. At the same time, it should be emphasized that in this study, as well as in other studies of this type under laboratory conditions, the initial densities of suspensions of the tested strains are many times higher than the level of contamination of touch surfaces under real conditions. In logarithmic terms, for the majority of the samples, a reduction in the initial bacterial suspension of at least three logarithms was obtained. When expressed as a percentage, for the majority of the strains tested, apart from ENT and CA, a reduction was obtained ranging from 95 to 100%.

The results acquired in our study are similar to the ones obtained by dos Santos and de Castro, who, in laboratory tests, achieved a 100% reduction in bacterial suspensions with a density of 10^8^ CFU/mL for strains from the species *S. aureus*, *S. epidermidis*, *E. coli*, *P. aeruginosa* and *S. enterica* and a reduction of four log for *C. auris*. When the tested device emitting UVC radiation was employed, Santos and de Castro observed an almost 100% reduction in the number of isolated CFUs [18].

The study conducted under clinical conditions should be treated as a pilot study due to the fact that it was limited to four patient rooms, from which swabs were taken from randomly selected surfaces before cleaning, after cleaning and then after disinfection. The swabs were used to assess microbiological contamination. The samples taken in each of the units gave rise to cultures of bacteria that are the natural flora of skin and the environment (air), as well as Gram-negative bacilli strains (*Acinetobacter* and *Pseudomonas*) and/or of the genus *Enterococcus*. These microorganisms are isolated from hospital-acquired infections, including those in intensive care units, and are often the cause of hospital outbreaks [19,20]. In this study, these strains were eliminated already at the cleaning stage, and the disinfection process led to a further significant reduction in the total number of bacteria.

Cremers-Pijpers et al., confirmed the effectiveness of UVC disinfection for handled electronic devices in a study carried out in two hospital units with a total of four-hundred DECT phones and smartphones, and in which the total reduction in bacteria contaminating the devices was 97.9% [21].

Ramos et al., after reviewing several studies in the field, also confirm the effectiveness of the UV-C light disinfecting, mostly as an adjunct to already existing terminal cleaning standard operating procedures [22]. According to these reviews, UV-Cs disinfecting even outperforms active hydrogen peroxide in the removal of MRSA, VRE and C. difficile and is especially useful in case of surfaces with a high microbial burden where there is frequent occupant use [22].

In infection control practice, the ultimate indicator of the correctness of infection prevention procedures or their effective implementation is the number of hospital-acquired infections. As for disinfection using non-contact technologies based on UV-C radiation, Raggi et al., assessed the clinical, operational and financial outcomes of this disinfection method concerning patient rooms and found significantly lower infection rates in the period when traditional methods were supplemented with this solution [23]. Additionally, the authors estimated the costs associated with additional hospitalization of patients with infections caused by selected multidrug-resistant strains and, for the period in which additional disinfection based on contactless UV-C technology was carried out, found a significant decrease in the average additional costs per patient from USD 1562.5 to USD 823.2 and in the total costs from USD 2,578,125.00 to USD 1,358,247.00. The study took into account MDRO strains of the species *Acinetobacter baumannii*, *Klebsiella pneumoniae*, *Staphylococcus aureus*, *Pseudomonas aeruginosa* and *Enterococcus faecalis*, that is, the species which we tested in terms of reduction effectiveness in this study.

The effectiveness of disinfection using UVC radiation has been confirmed not only in relation to bacteria of significant epidemiological importance, but also in relation to the SARS-CoV-2 virus. Criscuolo et al. [24] confirmed a reduction in the virus of over 99.9% in a period of 15 min on various types of surfaces, glass, plastic, gas, wood, wool and fleece, while Gidaro et al. [25] observed a reduction of over four log10 after 21–36 s, depending on the type of material that the media were made of: stainless steel, glass or plastic.

Olague et al. tested the effectiveness of eliminating the SARS-CoV-2 virus in laboratory conditions on various types of surfaces—polystyrene, polyvinyl chloride, stainless steel, MDF and VMDF. In every case, apart from MDF, elimination of viral particles of over 90% was confirmed, while for MDF, it was 19.9%. The time of exposure to UVC radiation was from 42 to 340 s [26].

The surface wettability results obtained are in line with the results in other works in which the authors widely report that there are correlations between surface wettability and the efficacy of microbial elimination by various materials; although, the exact mechanisms of mechanobactericidal action responsible for this are still not known and remain to be fully elucidated. These surfaces are usually designed and manufactured based upon nanotopologies found in nature, which are known to kill bacteria upon contact, as reported by Linklater [27]. These surfaces have, so far, been manufactured using materials such as silicon, titanium, stainless steel, glass, polymers and more. In particular, in his work, Valiei [28] reports that the bactericidal activity of etched silicon nanopillars on Pseudomonas aeruginosa was highest on superhydrophilic surfaces and decreased with increasing hydrophobicity. These findings suggest that superhydrophilic nanopillared surfaces are a superior choice for mechano-bactericidal activity, whereas superhydrophobic surfaces, although not bactericidal, may have antibiofouling properties through their self-cleaning effect. As previously mentioned, Linklater [28] reports the fabrication of superhydrophilic black silicon surfaces with well-defined surface geometries and wettability which are responsible for inactivating approx. 98% of *P. aeruginosa* cells. Wojcieszak [29] report that surface wettability of another important material exhibiting outstanding properties for medical applications—titanium doped with bioactive metals, i.e., copper or silver—may promote cell adhesion and an increase in the probability of copper ion migration from the film surface to the interior of microorganisms (due to penetration of cell membranes). Jalvo [30], on the other hand, reports that UV radiation can alter surface wettability and proves in his work that his smooth glass surfaces and glass microfiber filters with hydrophobic properties have become hydrophilic, and the antibacterial effect observed during tests using *Staphylococcus aureus* and *Pseudomonas putida* causes extensive membrane damage and significant production of intracellular reactive oxygen species in all TiO_2_-loaded irradiated specimens. The reduction in cell viability was over 99.9% (>3-log) for TiO_2_ on glass surfaces. The presented results of wettability in this paper prove the initial correlation of the bactericidal effectiveness of glass, plastic and steel, but in order to confirm it, they require continuation of research in the field of understanding the exact mechanism responsible for it.

## 5. Limitations of the Study

Our study confirms the effectiveness of reducing selected clinical bacteria strains as of special epidemiological importance, as well as reducing *C. auris* in laboratory conditions and in clinical wards. However, there are some limitations of this study, especially in case of the clinical part. In the laboratory part of this study, the limitation is the lack of repetitions for given strain, location and type of carrier. But the results are quite consistent, confirming that the experiment was run properly. In the hospital part, the limitation is the small number of samples in each hospital room, ranging from seven to eleven. Taking a swab from the surface before cleaning or disinfection caused a decrease in the number of bacterial, so the condition of swabbing after disinfection was not identical. Additionally, the total number of tests in the hospital was quite small, and we did not analyze the epidemiological situation in the hospital. At the time of tests, there were no outbreaks. That is why in the clinical part of our study should be taken as a pilot.

## Figures and Tables

**Table 1:** The effect of UV-C disinfection on bacterial suspension density depending on type of carrier.

Type of Carrier	Strain	Before Disinfection UV-C [CFU/mL]	Reduction after Disinfection UV-C [CFU/mL]		
			Mean	SD	In Total [%]
PLASTIC	SA	8.6 × 10^5^	8.59 × 10^5^	8.60 × 10^2^	99.9%
	KP	2.7 × 10^6^	2.7 × 10^6^	7.72 × 10^3^	99.9%
	AB	8.2 × 10^5^	8.06 × 10^5^	1.55 × 10^4^	98.3%
	ECLO	2.5 × 10^6^	2.53 × 10^6^	9.62 × 10^3^	99.7%
	ENT	9.0 × 10^5^	7.98 × 10^5^	1.50 × 10^5^	88.7%
	PAR	1.9 × 10^6^	1.92 × 10^6^	0.00	100.0%
	CA	8.0 × 10^4^	7.1 × 10^4^	8.13 × 10^3^	89.3%
	ALL STRAINS	1.40 × 10^6^	1.38 × 10^6^	1.00 × 10^6^	98.57%
GLASS	SA	9.0 × 10^5^	9.00 × 10^5^	8.94 × 10^1^	100.0%
	KP	2.2 × 10^6^	2.18 × 10^6^	1.31 × 10^3^	100.0%
	AB	1.2 × 10^6^	1.21 × 10^6^	7.80 × 10^3^	99.3%
	ECLO	3.0 × 10^6^	3.02 × 10^6^	4.10 × 10^3^	99.9%
	ENT	1.2 × 10^6^	1.14 × 10^6^	8.79 × 10^4^	95.0%
	PAR	2.1 × 10^6^	2.12 × 10^6^	1.79 × 10^2^	100.0%
	CA	8.0 × 10^4^	8.00 × 10^4^	8.94 × 10^1^	100.0%
	ALL STRAINS	1.53 × 10^6^	1.52 × 10^6^	9.78 × 10^5^	99.72%
STEEL	SA	1.1 × 10^6^	1.10 × 10^6^	1.79 × 10^2^	100.0%
	KP	2.0 × 10^6^	2.02 × 10^6^	1.41 × 10^3^	100.0%
	AB	5.2 × 10^5^	5.09 × 10^5^	1.30 × 10^4^	97.9%
	ECLO	2.3 × 10^6^	2.31 × 10^6^	7.13 × 10^3^	99.7%
	ENT	1.4 × 10^6^	1.35 × 10^6^	7.97 × 10^4^	96.6%
	PAR	2.4 × 10^6^	2.38 × 10^6^	7.16 × 10^2^	100.0%
	CA	4.0 × 10^4^	3.96 × 10^4^	8.76 × 10^2^	98.9%
	ALL STRAINS	1.44 × 10^6^	1.43 × 10^6^	9.81 × 10^5^	99.41%

Legend: CFU—colony-forming unit, SA—*Staphylococcus aureus*, KP—*Klebsiella pneumoniae*, AB—*Acinetobacter baumannii*, ECLO—*Enterobacter cloacae*, ENT—*Enterococcus faecalis*, PAR—*Pseudomonas aeruginosa*, CA—*Candida auris*.

**Table 2:** The effect of UV-C disinfection on bacterial suspension density reduction on plastic carrier depending on sample location.

	Strain	Before Disinfection UV-C [CFU/mL]	After Disinfection [CFU/mL (% of Reduction)]					
		-	No. 1	No. 2	No. 3	No. 4	No. 5	In Total
PLASTIC	SA	8.6 × 10^5^	2.0 × 10^2^ (99.98%)	4.0 × 10^2^ (99.95%)	1.0 × 10^3^ (99.88%)	1.0 × 10^3^ (99.88%)	2.4 × 10^3^ (99.72%)	99.9%
	KP	2.7 × 10^6^	0.0 (100%)	0.0 (100%)	4.0 × 10^2^ (99.99%)	2.0 × 10^2^ (99.99%)	1.7 × 10^4^ (99.36%)	99.9%
	AB *	8.2 × 10^5^	7.2 × 10^3^ (99.12%)	3.9 × 10^4^ (95.22%)	4.4 × 10^3^ (99.46%)	1.0 × 10^3^ (99.88%)	1.9 × 10^4^ (97.71%)	98.3%
	ECLO	2.5 × 10^6^	3.4 × 10^3^ (99.87%)	6.2 × 10^3^ (99.76%)	2.0 × 10^2^ (99.99%)	0.0 (100%)	2.3 × 10^4^ (99.09%)	99.7%
	ENT **	9.0 × 10^5^	8.0 × 10^4^ (91.11%)	3.6 × 10^4^ (86.00%)	2.0 × 10^4^ (97.78%)	8.0 × 10^3^ (99.11%)	3.7 × 10^5^ (59.33%)	88.7%
	PAR	1.9 × 10^6^	0.0 (100%)	0.0 (100%)	0.0 (100%)	0.0 (100%)	0.0 (100%)	100.0%
	CA	8.0 × 10^4^	6.0 × 10^2^ (99.25%)	1.9 × 10^4^ (76.25%)	1.2 × 10^4^ (85.50%)	0.0 (100%)	1.2 × 10^4^ (85.50%)	89.3%

Legend: CFU—colony-forming unit, SA—*Staphylococcus aureus*, KP—*Klebsiella pneumoniae*, AB—*Acinetobacter baumannii*, ECLO—*Enterobacter cloacae*, ENT—*Enterococcus faecalis*, PAR—*Pseudomonas aeruginosa*, CA—*Candida auris*. * Statistically significant differences between AB and PAR, KP *p* = 0.045; ** statistically significant differences between ENT and PAR, KP, SA, and ECLO *p* = 0.003.

**Table 3:** The effect of UV-C disinfection on bacterial suspension density reduction on glass carrier depending on sample location.

	Strain	Before Disinfection UV-C [CFU/mL]	After Disinfection [CFU/mL (% of Reduction)]					
		-	No. 1	No. 2	No. 3	No. 4	No. 5	In total
GLASS	SA	9.0 × 10^5^	2.0 × 10^2^ (99.98%)	0.0 (100%)	0.0 (100%)	0.0 (100%)	0.0 (100%)	100.0%
	KP	2.2 × 10^6^	3.0 × 10^3^ (99.86%)	2.0 × 10^2^ (99.99%)	0.0 (100%)	2.0 × 10^2^ (99.99%)	1.8 × 10^3^ (99.92%)	100.0%
	AB *	1.2 × 10^6^	1.0 × 10^3^ (99.92%)	8.0 × 10^3^ (99.34%)	1.9 × 10^4^ (98.48%)	2.0 × 10^2^ (99.98%)	1.3 × 10^4^ (98.97%)	99.3%
	ECLO	3.0 × 10^6^	1.2 × 10^3^ (99.96%)	2.0 × 10^2^ (99.99%)	1.6 × 10^3^ (99.95%)	0.0 (100%)	9.8 × 10^3^ (99.68%)	99.9%
	ENT **	1.2 × 10^6^	2.0 × 10^4^ (98.33%)	3.6 × 10^4^ (97.00%)	2.0 × 10^4^ (98.33%)	7.4 × 10^3^ (99.38%)	2.2 × 10^5^ (82.00%)	95.0%
	PAR	2.1 × 10^6^	0.0 (100%)	0.0 (100%)	0.0 (100%)	0.0 (100%)	4.0 × 10^2^ (99.98%)	100.0%
	CA	8.0 × 10^4^	0.0 (100%)	0.0 (100%)	0.0 (100%)	0.0 (100%)	2.0 × 10^2^ (99.75%)	100.0%

Legend: CFU—colony-forming unit, SA—*Staphylococcus aureus*, KP—*Klebsiella pneumoniae*, AB—*Acinetobacter baumannii*, ECLO—*Enterobacter cloacae*, ENT—*Enterococcus faecalis*, PAR—*Pseudomonas aeruginosa*, CA—*Candida auris*. * Statistically significant differences between AB and SA, CA, and PAR *p* = 0.008; ** statistically significant differences between ENT and SA, CA, PAR, and KP *p* < 0.001.

**Table 4:** The effect of UV-C disinfection on bacterial suspension density reduction on stainless steel carrier depending on sample location.

	Strain	Before Disinfection UV-C [CFU/mL]	After Disinfection [CFU/mL (% of Reduction)]					
		-	No. 1	No. 2	No. 3	No. 4	No. 5	In total
STEEL	SA	1.1 × 10^6^	4.0 × 10^2^ (99.96%)	2.0 × 10^2^ (99.98%)	0.0 (100%)	0.0 (100%)	0.0 (100%)	100.0%
	KP	2.0 × 10^6^	0.0 (100%)	0.0 (100%)	1.4 × 10^3^ (99.93%)	0.0 (100%)	3.2 × 10^3^ (99.84%)	100.0%
	AB	5.2 × 10^5^	0.0 (100%)	2.2 × 10^4^ (95.69%)	4.2 × 10^3^ (99.19%)	0.0 (100%)	2.7 × 10^4^ (94.81%)	97.9%
	ECLO	2.3 × 10^5^	8.6 × 10^3^ (99.63%)	1.6 × 10^4^ (99.33%)	0.0 (100%)	0.0 (100%)	1.2 × 10^4^ (99.47%)	99.7%
	ENT *	1.4 × 10^6^	2.3 × 10^4^ (98.34%)	1.5 × 10^4^ (98.96%)	1.4 × 10^4^ (99.03%)	0.0 (100%)	1.9 × 10^5^ (86.43%)	96.6%
	PAR	2.4 × 10^6^	0.0 (100%)	0.0 (100%)	0.0 (100%)	0.0 (100%)	1.6 × 10^3^ (99.93%)	100.0%
	CA	4.0 × 10^4^	0.0 (100%)	0.0 (100%)	0.0 (100%)	2.0 × 10^2^ (99.50%)	2.0 × 10^3^ (95.00%)	98.9%

Legend: CFU—colony-forming unit, SA—*Staphylococcus aureus*, KP—*Klebsiella pneumoniae*, AB—*Acinetobacter baumannii*, ECLO—*Enterobacter cloacae*, ENT—*Enterococcus faecalis*, PAR—*Pseudomonas aeruginosa*, CA—*Candida auris*. * Statistically significant differences between ENT and PAR, SA, CA, and KP *p* = 0.025.

**Table 5:** Effectiveness of UV-C disinfection in hospital wards, preceded and not-preceded by cleaning.

Before Decontamination (Cleaning or Disinfection)			After Cleaning			After Disinfection (Preceded or Not by Cleaning)		
No. of Positive/Negative Samples	No. of CFU	Bacteria Species	No. of Positive/Negative Samples	No. of CFU/Reduction Level	Bacteria species	No. of Positive/Negative Samples	No. of CFU/Reduction Level	Bacteria Species
**Time of Disinfection; Ward; Surfaces**								
**5 min 31 s;** Vascular surgery; bad frame, light switch, drip frame, toilet desk, shower curtain, washbasin tap, handle, mirror								
8/1	320	*S. epidermidis* *S. haemolyticus* *S. hominis* *S. warneri* *E. faecium* *Pseudomonas aeruginosa*	5/4	162 49.38%	*S. epidermidis* *S. hominis* *S. warneri* *S. saprophyticus* *Bacillus circulans* *Bacillus cereus*	1/8	5 98.44%	*S. warnerii*
**24 min 25 s;** Ist Anaestesiology and Intensive care (HTX) (ICU I); light switch, respirator frame, infusion pomp, handle, resuscitation trolley handle, door glass, handle, washbasin tap, light switch, floor								
9/2	105	*S. haemolyticus* *S. pasteuri* *E. faecalis* *Bacillus simplex* *Bacillus pumilus* *Bacillus cereus* *Paenibacillus amylolyticus* *Glutamicibacter ceratinolyticus* *Pseudomonas fulva*	8/3	72 31.43%	*S. haemolyticus* *S. cohnii* *Bacillus simplex* *Bacillus circulans* *Brevundimonas diminuta* *Pseudomonas oryzihabitans*	2/9	4 96.19%	*S. saprophiticus* *S. cohnii* *Brevundimonas diminuta*
**6 min;** IIIrd Anesthesiology and Intensive care (isolation room) (ICU III); basket flap, infusion pomp, drip frame, handle, glass, window, bad table								
5/2	120	*S. epidermidis* *S. haemolyticus* *S. hominis* *S. warneri* *Microbacterium paraoxydans* *Acinetobacter baumannii*	4/3	110 8.33%	*S. epidermidis* *S. haemolyticus* *S. hominis*	0/7	0 100%	
**11 min 41 s;** Ist Anesthesiology and Intensive care; EKG monitor, respirator monitor, bad table, resuscitation trolley, respirator handle, resuscitation trolley handles, trolley for the gowns, glass door, handle, EKG machine								
7/3	181	*S. epidermidis* *S. haemolyticus* *S. hominis* *E. faecalis* *Bacillus megaterium* *Bacillus flexus* *Bacillus mycoides*	3/7	18 90.06%	*S. epidermidis* *S. haemolyticus* *Bacillus subtilis*	0/10	0 100%	
**4 min;** Cardiosurgery; bed frame, light switch, bed table, matress pump, oxygen bottle switch, drip frame, handle, handle of bad table, stetoscope								
9/10	213	*S. epidermidis* *S. haemolyticus* *S. warneri* *Corynebacterium pseudodiphtericum* *E. faecalis*	Not done	Not done	Not done	3/10	10 95.3%	*S. epidermidis* *S. hominis* *Micrococcus luteus*
**9 min 27 s;** Heart, Vascular Surgery and Transplantology recovery room; drip frame, handle, balcony handle, light switch, cardiomonitor, toilet desk, flush button, washbasin tap, handle, mirror								
10/11	362	*S. epidermidis* *S. haemolyticus* *S. hominis* *S. capitis* *E. faecalis* *Bacillus clausii* *Streptomyces badius* *Corynebacterium amycolatum*	Not done	Not done	Not done	2/11	72 80.1%	*S. haemolyticus* *Bacillus circulans*

**Table 6:** Results of contact angle measurements of surface of tested materials with 2 µL demineralized water at a temperature of 20 °C and relative humidity of 50%.

Test Material	Droplet Volume	Contact Angle	Wettability Regime	Temperature	Humidity	Evaporation Time	Evaporation Time
	[µL]	[°]		[°C]	[%]	[s]	[min.]
Glass	2.0	19.9	moderately superhydrophilic	20	50	752	13
Steel		51.1	hydrophilic			1950	33
Plastic		90.3	hydrophobic			3382	56

## Data Availability

Original data are available at corresponding author upon reasonable request.
